# A medial pivot kinematic pattern could be achieved in a significant proportion of patients with varus knees after primary total knee arthroplasty using functional positioning

**DOI:** 10.1002/jeo2.70343

**Published:** 2025-07-07

**Authors:** Fabio Mancino, Andreas Fontalis, Ricci Plastow, Fares S. Haddad

**Affiliations:** ^1^ Department of Trauma and Orthopaedic Surgery University College Hospital London UK; ^2^ The Princess Grace Hospital University College London Hospitals London UK; ^3^ Orthopaedic Department University College London Hospitals London UK

**Keywords:** kinematics, medial pivot, robotic, total knee arthroplasty

## Abstract

**Purpose:**

Physiological knee kinematics typically exhibit a medial pivot (MP) pattern and femoral rollback during flexion. There is a significant gap in the literature pertaining to the evaluation of kinematic patterns following total knee arthroplasty (TKA). The aim of this study was to evaluate the proportion of patients achieving a MP kinematic pattern following robotic‐arm‐assisted TKA using functional positioning.

**Methods:**

Sixty consecutive patients who underwent primary robotic‐assisted TKA employing functional positioning were investigated. Following final positioning, sensor‐embedded tibial trials were used to assess the kinematic pattern. Two senior fellowship‐trained surgeons documented the kinematic tracking of the tibiofemoral articulation and centre of load on the sensor display monitor. The differential load pressure was captured intraoperatively at 10°, 45° and 90° to substantiate the kinematic pattern. Significance was for *p*‐value < 0.05.

**Results:**

A MP kinematic pattern was observed in 29 patients (48%). These patients exhibited a median medial load of 21.0 lbf at 10°, 15 lbf at 45° and 13 lbf at 90°, while the median lateral load was 13 lbf at 10°, 8 lbf at 45° and 6 lbf at 90°. The patients with MP kinematics were associated with significantly greater medial measured loads at 10° flexion (21 vs. 18 lbf; *p* < 0.001) and with lower measured loads in the lateral compartment at 10° (13 vs. 15 lbf; *p* = 0.011), 45° (8 vs. 13 lbf; *p* = 0.011) and 90° of flexion (6 vs. 10 lbf; *p *< 0.001). Mean differential load pressure between the compartments was <15 lbf at 10°, 45° and 90° in both groups.

**Conclusion:**

In our cohort, using robotic arm assistance with functional positioning, we observed a MP kinematic pattern in approximately 50% of the cases. Further longer‐term studies are warranted to evaluate the impact of the kinematic pattern on clinical and patient‐reported outcomes.

**Level of Evidence:**

Level III, retrospective cohort study.

**Abbreviations**
AP: anteroposterior
CR: cruciate retaining
CT: computerised tomography
FB: fixed bearing
fMA: femoral mechanical axis
HIPAA: health insurance portability and accountability act
HKA: hip knee ankle angle
IRB: institutional review board
lbf: pound force
MP: medial pivot
OA: osteoarthritis
PS: posterior stabilised
ROM: range of motion
SD: standard deviation
TEA: transepicondylar axis
TKA: total knee arthroplasty
tMA: tibial mechanical axis
UC: ultra congruent

## INTRODUCTION

Total knee arthroplasty (TKA) is a well‐established intervention, yielding substantial improvements in pain relief and functional mobility. However, there remains a proportion of patients between 10% and 20%, who are dissatisfied with the outcomes [25].

Possible contributors to these suboptimal subjective outcomes include age, obesity, complications and an abnormal knee kinematic pattern [4].

Dynamic kinematics in normal knees exhibit medial pivot (MP) motion, with the lateral femoral condyle sliding posteriorly on the tibial plateau during flexion, while a pivoting movement is observed in the medial compartment [7]. The anatomy of the tibial plateau allows for this observed motion and creates a knee that is inherently more stable on the medial side [20, 38]. However, large variations in knee kinematics have been reported after TKA [6, 16]. The drive to replicate native knee kinematics is partly due to the limitations of current implant designs, which often fail to restore natural knee motion, potentially leading to patient dissatisfaction [8, 42]. Nevertheless, due to the lack of readily accessible tools for quantifying kinematics, there is a notable lack of literature on the evaluation of different kinematic patterns following TKA.

Furthermore, a growing body of evidence suggests that this ‘one‐size‐fits‐all’ philosophy often overlooks the considerable differences in the unique native alignment of each individual [22], potentially forcing the knee into unnatural kinematics [35]. A paradigm shift towards re‐establishing the patient's prearthritic (constitutional) alignment has been suggested, as a potential avenue to achieve more natural knee kinematics [1] and soft tissue balance [12, 23]. Individualised alignment strategies tailored to the individual's unique biomechanics, such as functional alignment and positioning, have shown promising results [3, 10, 18, 29, 32, 37].

Pressure sensors are disposable tibial inserts with embedded microelectronic detectors. They provide real‐time dynamic pressure readings along with data on the tibiofemoral position and peak load centres in the medial and lateral compartments [5]. Surgeons can evaluate the pressure loads and kinematic tracking throughout the range of motion (ROM), using a digital interface. This in vivo feedback enables adjustments in line with the specific alignment philosophy principles. These devices have been successfully used to intraoperatively identify medio‐lateral overload and instability, especially at mid‐flexion and to personalise the individual kinematic patterns [5, 13, 27].

The aim of this study was to evaluate the proportion of patients achieving a MP kinematic pattern following robotic‐arm assisted TKA with a contemporary implant design, using functional alignment.

## METHODS

This is a single surgeon retrospective study of prospectively collected consecutive patients who underwent robotic‐arm‐assisted primary TKA in 2022. The inclusion criteria were patients >18 years of age with advanced primary knee osteoarthritis (OA) grade 3 or 4 according to the Kellgren–Lawrence classification [19] not responsive to conservative treatment, and with either a neutral alignment or varus deformity less than 15° on the mechanical axis. The exclusion criteria were severe varus alignment >15°, valgus alignment, multidirectional knee instability requiring a higher level of constraint than a posterior stabilised (PS) liner, previous knee surgery affecting the overall lower limb alignment (e.g. osteotomy) or knee stability (e.g. anterior cruciate ligament reconstruction), posttraumatic OA, or inability to communicate in the English language.

All study patients underwent preoperative computerised tomography (CT) scans of the knee joint to detail osseous anatomy, identify bone resection landmarks, assess bone defects and plan implant positioning and sizes. The femoral mechanical axis (fMA) was defined as a line extending from the centre of the femoral head to the top of the femoral notch. The tibial mechanical axis (tMA) was defined as the line from the tibia midpoint at the knee joint (centre of the tibial spines) to the centre of the tibial plafond at the ankle. The Hip–Knee–Ankle angle (HKA) was defined as the medial angle between the fMA and the tMA. All procedures were performed by the senior author using the principles of functional alignment. The initial bone preparation and component positioning plan aimed to achieve neutral alignment (HKA = 180°). This was adjusted intraoperatively to restore the plane and obliquity of the joint as dictated by the soft tissues within the boundaries of 6° varus and 3° valgus (174° ≤ HKA ≤ 183°). The femoral component was initially planned perpendicular to the mechanical axis of the femur and parallel to the transepicondylar axis (TEA). Femoral component size and flexion were selected to achieve maximal bone coverage while preventing any overhang or notching. The tibial component was also planned perpendicular to the mechanical axis of the tibia with a posterior slope of 1°–3°. The combined femoral flexion and tibial posterior slope was kept within the boundary of 10° as per recommendations of the company. Tibial implant size was planned to achieve maximum mediolateral coverage without any overhang.

All operative procedures were performed using the standard medial parapatellar approach. Intraincisional registration pins were placed in the femur and tibia and fixed infrared rays mounted onto these to enable motion capture tracking. Registration points were taken on osseous landmarks. Any femoral and tibial osteophytes were excised. Intraoperative medial and lateral joint gaps in knee flexion and extension were initially assessed and used to adjust the intraoperative plan to achieve symmetrical flexion–extension gaps across medial and lateral compartments, targeting a laxity range of 19–21 mm (Mako software 1.0), with an acceptable difference of ±2 mm between the compartments [14]. A slightly laxer gap in the lateral compartment during flexion was considered acceptable to facilitate MP kinematics. The RIO Robotic Interactive Orthopaedic system (Stryker Corp, MAKO Surgical Corp) was used to execute the planned distal femoral and proximal tibial bone resections. Intraoperative medial and lateral joint gaps were then rechecked with gap balancing instruments and used to fine‐tune component positions to achieve the desired limb alignment, balanced flexion–extension gaps and ROM.

Implant positions were manipulated in all three planes to restore the plane and obliquity of the joint line as dictated by the periarticular soft tissues, while limiting any soft tissue releases. The patella was resurfaced in all cases. The cemented Triathlon® PS implant (Stryker) knee system with an asymmetrical patellar resurfacing button was used in all study patients. The Triathlon® Knee System Universal Baseplate was used in every case, encompassing a 20 mm bullet tip at the bottom of the tibial keel.

The Verasense™ tibial trial insert (former OrthoSensorTM; Stryker Corp) was used to assess the knee balance and kinematics both with the trial components and the definitive components before the final polyethene insert. The load measurements were collected at 10°, 45° and 90° of flexion with the patella relocated in the trochlear groove and the capsule partially closed by three single sutures [36]. A passive ROM from maximum extension to maximum flexion was performed. The flexion movement began by supporting the foot posteriorly with an open palm to record the full extension position. While supporting the foot, the surgeon used his opposite hand to lift the thigh gently, flexing the hip and knee [31]. The numbers were recorded at each flexion pose. A knee was considered stable and balanced if the medial load pressure was < 50 pound force (lbf), the lateral below 40 lbf and the mean difference between the two compartments' loads was within 15 lbf. A MP kinematic pattern was considered with an average mediolateral intracompartment pressure difference between 5 and 15 lbf in favour of the medial compartment [5] and with a pattern of a stable, medial centre of load and a shifting lateral centre of load in the anteroposterior (AP) plane. In the case of an intracompartment pressure difference <5 or in favour of the lateral compartment, it was considered a non‐MP kinematic pattern. The kinematic pattern was evaluated twice: intraoperatively by the operating surgeon and postoperatively by a second, adult reconstruction fellowship‐trained arthroplasty surgeon using the saved images. Other kinematic patterns were classified as non‐MP.

The study was performed in accordance with the ethical standards in the 1964 Declaration of Helsinki and with the Health Insurance Portability and Accountability Act of 1996 (HIPAA) regulations. The Institutional Review Board (IRB) of the author's institution defined this study as exempt from IRB approval (retrospective study on a well‐established surgical procedure and commercialised implant). Each patient provided written consent for participation in the study prior to enrolment.

### Data analysis

Descriptive results are presented as mean, standard deviation (SD) or range for normally distributed variables, or as median and interquartile range (IQR) (Quartile 1, Quartile 3) for nonnormally distributed data. The normality of distributions was evaluated using the Kolmogorov–Smirnov and Shapiro–Wilk tests. Continuous variables were compared using the independent samples *t*‐test or the Mann–Whitney *U* test, depending on data distribution. Categorical variables were compared using the chi‐squared test or Fisher's exact test. Statistical significance was set at a *p*‐value < 0.05. All data were analysed using the SPSS Statistics software v. 29 (IBM).

## RESULTS

In this study, 60 patients undergoing primary robotic‐assisted TKA were included. The mean age was 68.5 ± 7.3 years, with 63% of the cohort being female (38 patients). The mean body mass index (BMI) was 28.9 kg/m^2^ and the right side was operated on in 55% of the cases. The median preoperative alignment was 173.5° (IQR 1, 3; 171.8°–177.0°).

Overall, the median measured loads in the medial compartment with the final components in place were 20 lbf at 10° flexion (IQR 1, 3; 16.8–24.0 lbf), 15 lbf at 45° flexion (IQR 1, 3; 11.8–17.0 lbf) and 13 lbf at 90° flexion (IQR 1, 3; 10.0–17.0 lbf). The median measured loads in the lateral compartment with the final components implanted were 14 lbf at 10° flexion (IQR 1, 3; 11–17 lbf), 11 lbf at 45° flexion (IQR 1, 3; 7.0–13.3 lbf) and 9.0 lbf at 90° flexion (IQR 1, 3; 6.0–12.0 lbf).

Among the patients, 48% (29 of 60 patients) exhibited MP kinematics. In these patients, the median measured loads in the medial compartment were 21.0 lbf at 10° flexion, 15.0 lbf at 45° flexion and 13.0 lbf at 90° flexion. The median measured loads in the lateral compartments were 13.0 lbf at 10° flexion, 8.0 lbf at 45° flexion and 6.0 lbf at 90° flexion (Table 1).

**Table 1:** Measured load pressures across the patient population.

Degrees of flexion	Medial (MP) lbf	Medial (non‐MP) lbf	*p*	Lateral (MP) lbf	Lateral (non‐MP) lbf	*p*
10° (Quartile 1, Quartile 3)	21.0 (19.0, 27.0)	18.0 (11.0, 20.0)	<0.001^a^	13.0 (10.0, 15.0)	15.0 (13.0, 18.5)	0.006^a^
45° (Quartile 1, Quartile 3)	15.0 (11.0, 19.0)	15.0 (12.0, 17.0)	0.953^b^	8.0 (5.0, 13.0)	13.0 (8.5, 14.0)	0.011^b^
90° (Quartile 1, Quartile 3)	13.0 (10.0, 18.0)	13.0 (9.0, 16.0)	0.291^b^	6.0 (4.0, 11.0)	10.0 (9.0, 13.0)	<0.001^a^

Abbreviations: lbf, pound force; MP, medial pivot.

a Student's *t*‐test.

^b^ Mann–Whitney *U*.

**Figure 1:**
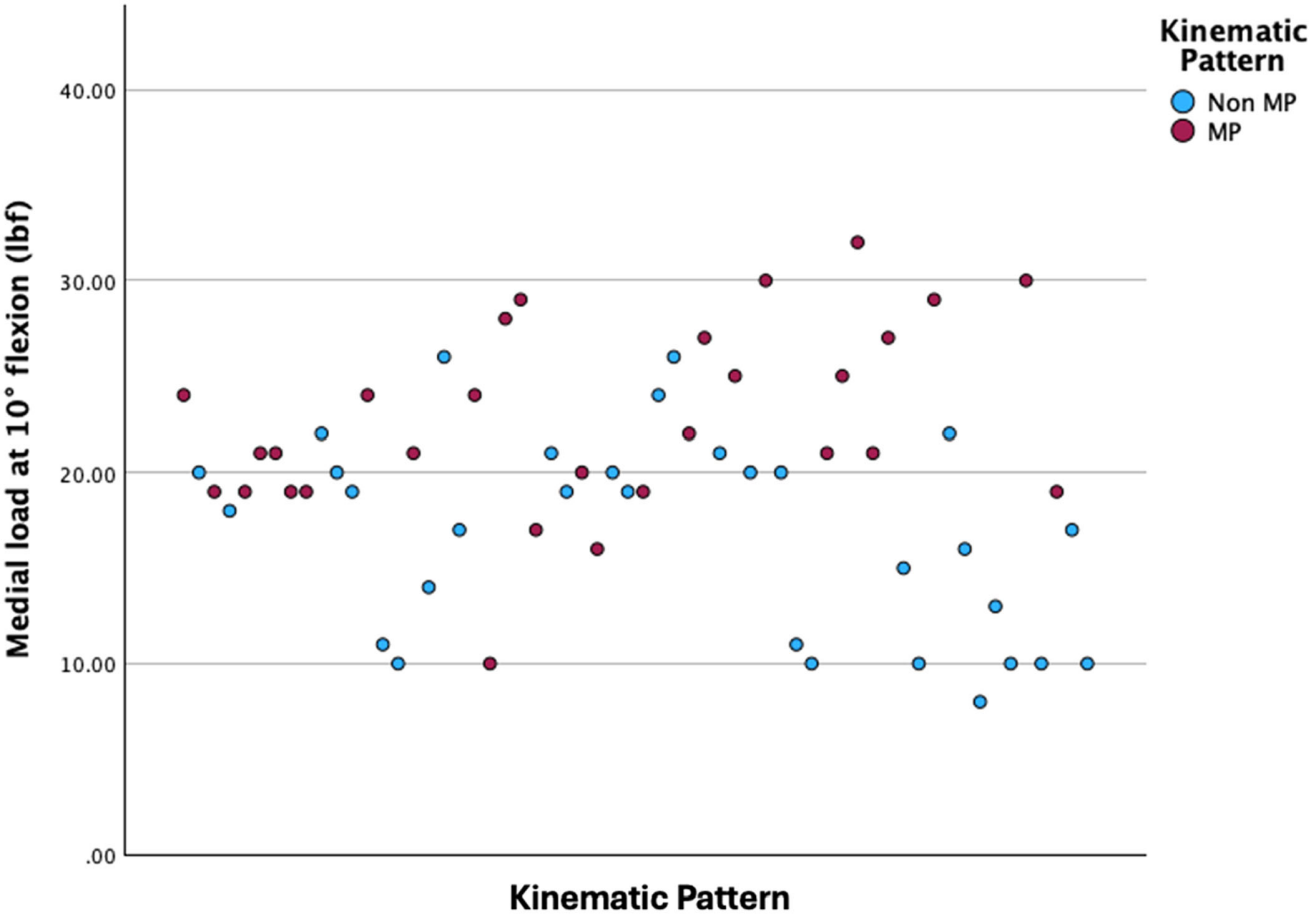
Scatter plot depicting the measured loads in the medial compartment at 10° flexion. lbf, pound force; MP, medial pivot.

**Figure 2:**
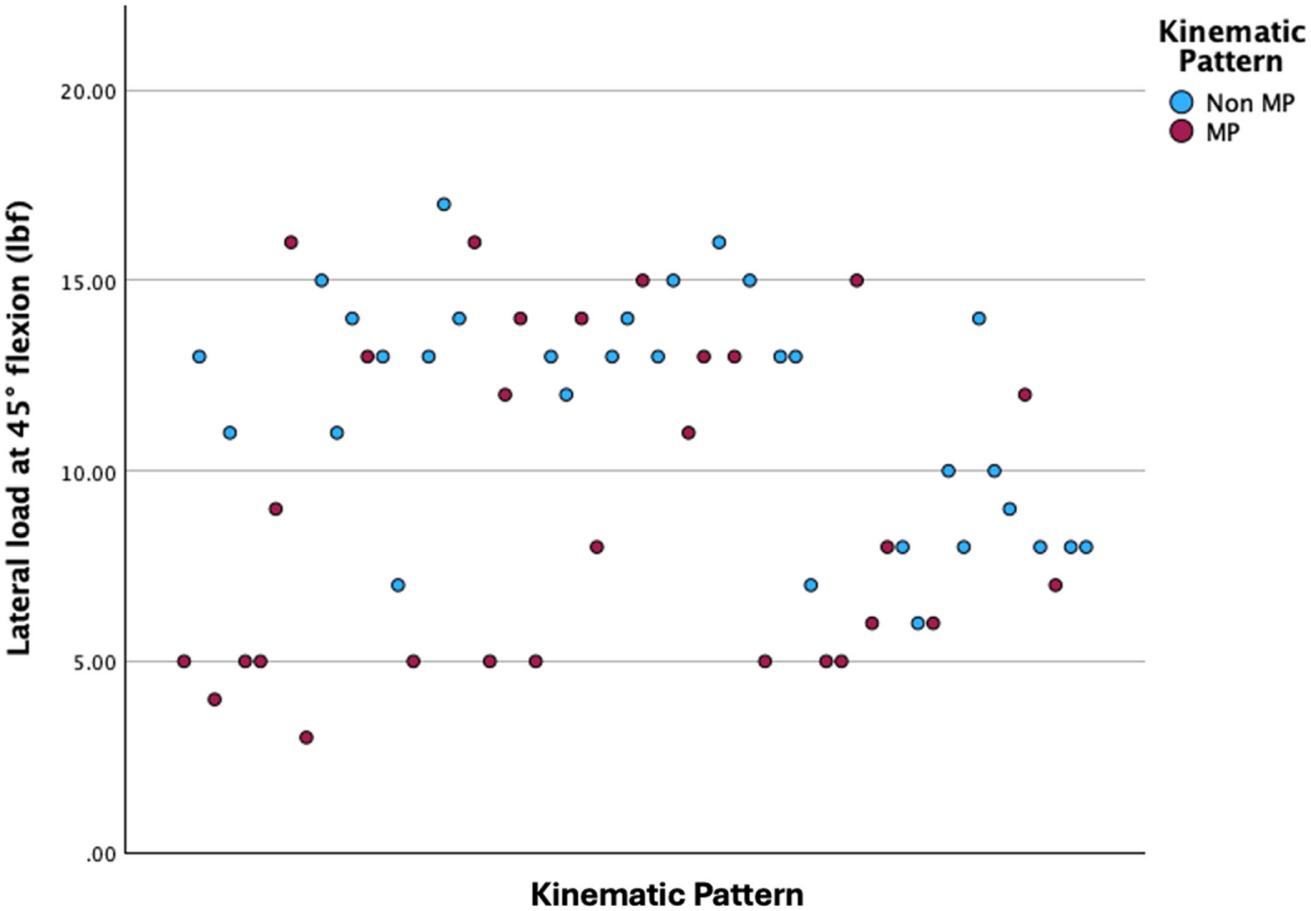
Scatter plot depicting the measured loads in the lateral compartment at 45° flexion. lbf, pound force; MP, medial pivot.

**Figure 3:**
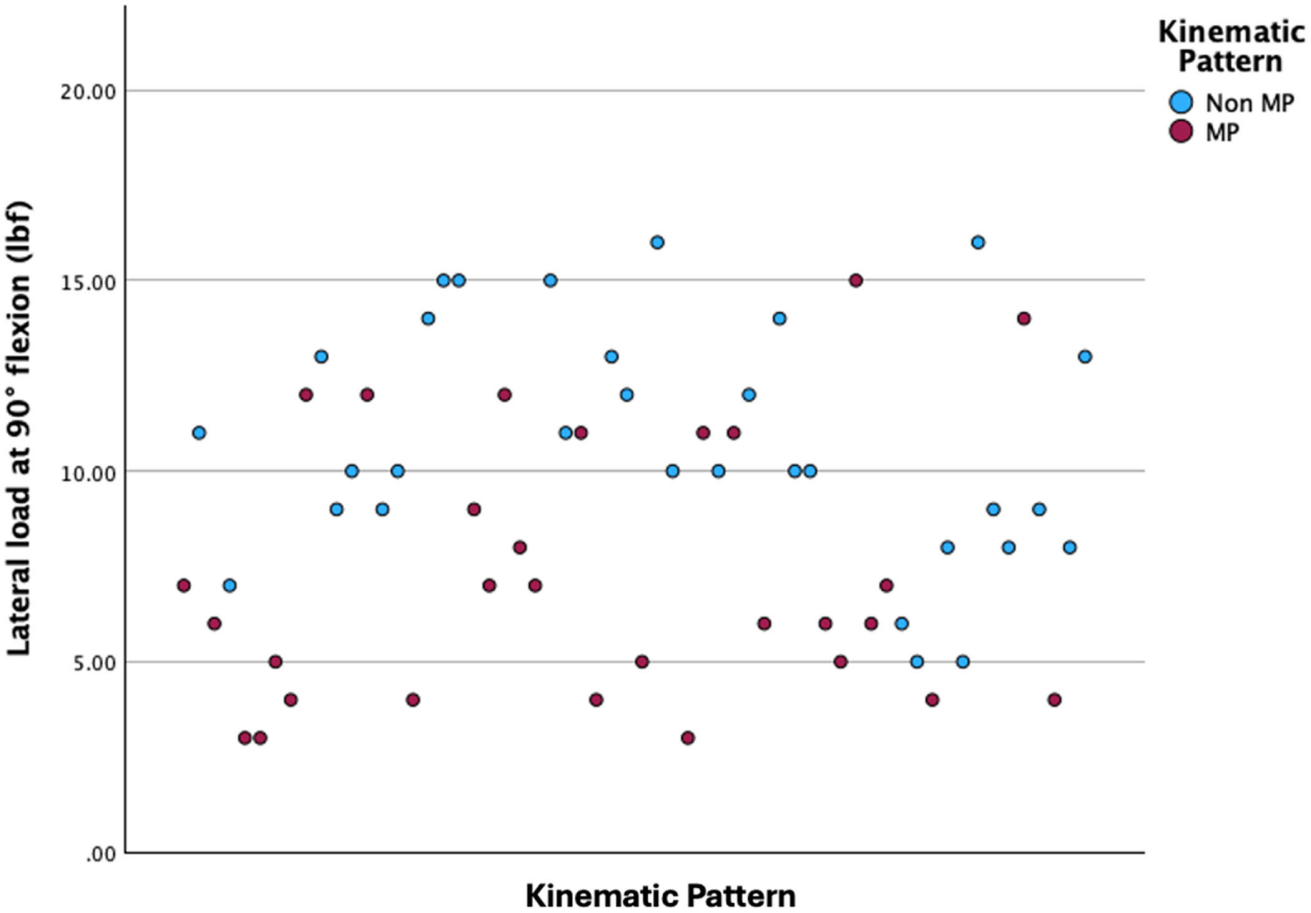
Scatter plot depicting the measured loads in the lateral compartment at 90° flexion. lbf, pound force; MP, medial pivot.

The patients with MP kinematics were associated with significantly greater medial measured loads at 10° flexion (21 vs. 18 lbf; Independent Samples Mann–Whitney *U*‐test, *p* < 0.001, Figure 1) and with lower measured loads in the lateral compartment at 10° flexion (13 vs. 15 lbf; Independent Samples Mann–Whitney *U*‐test, *p* = 0.011), 45° flexion (8 vs. 13 lbf; Independent Samples Mann–Whitney *U*‐test, *p* = 0.011, Figure 2) and 90° flexion (6 vs. 10 lbf; Independent Samples Mann–Whitney *U*‐test, *p* < 0.001, Figure 3).

Patients with a moderate varus native deformity (>10°) were more likely to show a MP kinematic pattern (8 of 10 patients; *χ* ^2^ *p* = 0.028).

Mean differential load pressure between the compartments was <15 lbf at 10°, 45° and 90° in both groups, corroborating mediolateral stability of the tibiofemoral articulation.

## DISCUSSION

This observational study showed that MP kinematics was achieved in a relatively high proportion of patients affected by mild‐to‐moderate varus OA using a modern fixed‐bearing (FB) PS knee insert, utilising functional positioning with robotic‐arm assistance. Intraoperative stability was confirmed at 10° and 90° of flexion. While the robotic software does not evaluate mid‐flexion stability directly, this parameter was indirectly evaluated through compartmental pressure measurements at 10°, 45° and 90° of flexion using the Verasense pressure sensor.

TKA designs do not invariably restore normal knee biomechanics. In a natural knee joint during deep flexion, the femur pivots medially relative to the tibia [11]. The lateral femoral condyle undergoes more pronounced AP motion, while the medial femoral condyle shows minimal rollback activity [7, 11, 15, 17, 30]. Research focused on restoring natural knee movement has advanced our understanding of how prosthetic design influences knee function, leading to the development of modern implants. The single‐radius femoral design has been shown to maintain collateral isometry throughout the arc of motion [39]. In addition, the Triathlon PS Knee System is characterised by an inner box width of 16.2 mm and box height of 20.5 mm with an associated insert that allows for a ±20° internal/external rotation.

Intraoperative load compartment sensors offer a quantitative method to assess soft tissue balancing and component alignment. They provide objective, real‐time data on tibiofemoral alignment and pressure distribution at peak contact points in the medial and lateral compartments [13]. It has been suggested that digital sensor data can provide more accurate and reliable assessments of knee soft tissue balancing at 10°, 45° and 90° [21], than an experienced surgeon. Erard et al. [9] reported a 94% restoration of a well‐balanced knee at 10° and 90° of flexion in patients undergoing image‐based robotic‐assisted TKA with varus deformity.

In this study, we observed a MP pattern in 48% of the patients. Warth et al. [40] reported an overall 40% MP kinematic pattern in a retrospective analysis of mechanically aligned Triathlon Knee System TKAs using both cruciate retaining (CR) and PS inserts in combination with the Verasense sensor‐embedded tibial trial. In‐vivo studies have indicated similar MP kinematics between PS and ultra‐congruent (UC) inserts despite UC designs offering greater anteroposterior stability, resulting in less femoral rollback [34]. A similar kinematic pattern has been reported during activities of daily living using a FB PS design with 60% exhibiting medial pivoting during sit‐to‐stand movements [2, 43]. These findings highlight the impact of prosthetic design alongside the alignment technique and soft tissue balancing on postoperative joint kinematics. The medial femoral–tibial compartment and the postcam mechanism influence the kinematics in a FB PS design. The postcam mechanism can avoid paradoxical translations, especially in deep‐ and mid‐flexion. Moreover, it has been reported that CT‐based robotic TKA is associated with consistent, equal load distribution between the medial and lateral compartments through the functional arc of motion [26, 27].

The influence of MP kinematics on postoperative clinical outcomes remains a topic of ongoing discussion. It has been shown that restoring MP kinematics using a PS implant design in patients with medial OA is associated with improved outcomes in activities and satisfaction at a minimum 24‐month follow‐up, including a greater deep flexion angle [31]. Similarly, Warth et al. [40] observed a trend towards improved median Knee Society Objective scores with a MP pattern using PS inserts, despite not reaching significance at the 1‐year mark. Additionally, Pizza et al. [33] showed that AP translations of the medial compartment in a FB PS TKA are associated with postoperative clinical outcomes. Identifying patients likely to achieve a MP pattern after PS TKA could eliminate the need for specific ball‐and‐socket design implants aimed at restoring native kinematics and improving postoperative outcomes.

Questions persist about why different kinematic patterns are observed among patients with comparable baseline characteristics, surgical techniques and prosthetic implants. It could be hypothesised that the kinematic pattern is associated with the starting deformity, with a greater varus deformity noted in patients exhibiting MP movement. This is in discordance with the study by Nishio et al. [31] where the patients with a nonmedial‐pivot kinematics were associated with a greater varus deformity while the different kinematics was possibly ascribed to the amount of soft tissue release performed to obtain equal gap balance. However, the small sample size warrants caution when interpreting these results. In line with the functional alignment principles, soft tissue release was not performed, aiming for a slightly looser lateral compartment, particularly during flexion. In fact, the lateral compartment was looser at 45° and 90° compared to 10°. Therefore, one possible explanation could be that the variability of a tighter medial compartment coupled with a looser lateral compartment, alongside a single radius PS design, may contribute to a MP kinematic pattern.

There are several limitations to this study. First, the assessment of the knee kinematics was performed intraoperatively and not preoperatively; thus, the baseline kinematic pattern of the patients is unknown. However, it is important to note that assessing the kinematics of a pathological arthritic knee, which may not represent those of a healthy native knee, was not the aim of this study. Second, an inherent limitation of the sensor is the positioning of the foot in terms of internal/external rotation and the intraoperative loads detected could be influenced by varus or valgus stress applied to the knee. To limit this source of bias, the senior author conducted all load pressure measurements to reduce variability. Third, the clinical relevance of the present study is yet to be determined despite the promising results of achieving a MP in approximately half of the patients. Fourth, load measurements were not taken under weight‐bearing conditions, unlike the normal gait circumstances; however, it has been shown that passive intraoperative load registration can correlate with postoperative knee kinematics [24, 41]. Lastly, there could be limitations associated with the utilisation of the Verasense sensor trial, which has been discontinued from the market, with a PS implant design. However, this has been validated in previous similar studies [28, 36, 40].

## CONCLUSION

In conclusion, understanding and restoring physiological knee kinematics could play a pivotal role in achieving optimal outcomes and patient satisfaction after primary TKA. In case of a mild to moderate varus deformity, MP kinematics can be achieved in almost half of the patients using a single‐radius modern PS implant adhering to the functional alignment principles. Further research is required to validate these preliminary findings and to better determine which patients are more likely to have physiological knee kinematics restored with a PS implant.

## AUTHOR CONTRIBUTIONS

All authors contributed to the study. Conceptualisation was performed by Fares S. Haddad. Material preparation and data collection were performed by Fabio Mancino and Andreas Fontalis. Data analysis was performed by Fabio Mancino and Andreas Fontalis. The draft of the manuscript was written by Fabio Mancino and Andreas Fontalis. Ricci Plastow and FSH commented on previous versions. Supervision and final approval was under Fares S. Haddad.

## CONFLICT OF INTEREST STATEMENT

Prof. Fares S. Haddad reports the following: BOSTAA (Board or committee member), British Orthopaedic Association (Board or committee member), Corin (IP royalties), Journal of Bone and Joint Surgery—British (Editorial or governing board), Matortho (IP royalties), Orthopedics Today (Editorial or governing board), Smith & Nephew (IP royalties; Paid consultant; Research support), Stryker (IP royalties; Paid consultant; Research support). All the authors declare they have no financial interests directly or indirectly related to the study that would wrongly influence the outcomes of the study. No benefits in any form have been received or will be received from a commercial party related directly or indirectly to the subject of this article.

## ETHICS STATEMENT

The study was performed in accordance with the ethical standards in the 1964 Declaration of Helsinki and with the Health Insurance Portability and Accountability Act of 1996 (HIPAA) regulations. The Institutional Review Board (IRB) of the author's institution defined this study as exempt from IRB approval (retrospective study on a well‐established surgical procedure and commercialised implant). Each patient provided written consent for participation in the study prior to enrolment.

## Data Availability

Data are available under reasonable request.
